# Physiological Electrical Signals Promote Chain Migration of Neuroblasts by Up-Regulating P2Y1 Purinergic Receptors and Enhancing Cell Adhesion

**DOI:** 10.1007/s12015-014-9524-1

**Published:** 2014-08-07

**Authors:** Lin Cao, Jin Pu, Roderick H. Scott, Jared Ching, Colin D. McCaig

**Affiliations:** 1 School of Medical Sciences, Institute of Medical Sciences, University of Aberdeen, Aberdeen, AB25 2ZD UK; 2 Department of Neurosurgery, Aberdeen Royal Infirmary, Aberdeen, AB25 2ZD UK

**Keywords:** Neuroblasts, Chain migration, P2Y1, β-catenin, Extracellular electrical gradients

## Abstract

Neuroblasts migrate as directed chains of cells during development and following brain damage. A fuller understanding of the mechanisms driving this will help define its developmental significance and in the refinement of strategies for brain repair using transplanted stem cells. Recently, we reported that in adult mouse there are ionic gradients within the extracellular spaces that create an electrical field (EF) within the rostral migratory stream (RMS), and that this acts as a guidance cue for neuroblast migration. Here, we demonstrate an endogenous EF in brain slices and show that mimicking this by applying an EF of physiological strength, switches on chain migration in mouse neurospheres and in the SH-SY5Y neuroblastoma cell line. Firstly, we detected a substantial endogenous EF of 31.8 ± 4.5 mV/mm using microelectrode recordings from explants of the subventricular zone (SVZ). Pharmacological inhibition of this EF, effectively blocked chain migration in 3D cultures of SVZ explants. To mimic this EF, we applied a physiological EF and found that this increased the expression of N-cadherin and β-catenin, both of which promote cell-cell adhesion. Intriguingly, we found that the EF up-regulated P2Y purinoceptor 1 (P2Y1) to contribute to chain migration of neuroblasts through regulating the expression of N-cadherin, β-catenin and the activation of PKC. Our results indicate that the naturally occurring EF in brain serves as a novel stimulant and directional guidance cue for neuronal chain migration, via up-regulation of P2Y1.

**Electronic supplementary material:**

The online version of this article (doi:10.1007/s12015-014-9524-1) contains supplementary material, which is available to authorized users.

## Introduction

The migration of neuronal precursor cells is essential in CNS development and repair. In postnatal mammalian brain, neuroblasts from the subventricular zone (SVZ) migrate within the rostral migratory stream (RMS) in intimate cell-cell contact with one another, forming a chain-like organization without a glial scaffold [1–3].

The persistence of chain migration depends on the interplay of directional cell movement and biased cell to cell contact [4]. For example, cell–cell interactions mediated via selective adhesion molecules establish and maintain chain migration. N-cadherin is expressed abundantly in chain migrating cells in the SVZ and RMS, but is down-regulated after cells exit these regions [5]. Another cell adhesion molecule, NCAM, is expressed at high levels on neuronal precursor cells migrating towards the olfactory bulb (OB) [6, 7] and girdin, α6β1 integrin and miR-9 (brain-specific microRNA-9) are additional intrinsic factors regulating chain migration along the RMS [8–10]. However, it remains poorly understood how neuroblasts use locally acting extracellular cues and intrinsic molecular machinery to coordinate their behaviour during chain migration within the RMS [8].

Extracellular electrical signals also regulate neuronal migration, nerve growth cone guidance, nerve sprouting and nerve regeneration and are widespread in developing and regenerating tissues, including brain and spinal cord [11, 12]. They are present during neurulation, are expressed continuously across the wall of the differentiating neural tube and disrupting them, by chemical, physical, or electrical means causes major developmental abnormalities in the formation of the nervous system [13–18]. In addition, epileptic seizure, stroke, ischaemia, migraine and acute damage to the hippocampus all induce extracellular electrical signals in brain that persist for hours to days [19–23]. Recently, we measured directly an electrical gradient of 5.7 ± 1.2 mV/mm within the extracellular spaces along the RMS ex vivo [24]. Using an applied EF of similar strength (~5 mV/mm), directional migration of epithelial cells, neonatal neurones and neuroblasts is induced in vitro and in vivo [24–28].

Neuronal chain migration involves coordinated cell migrations. Collective migration keeps tissues intact during remodelling, allows mobile cells to carry immobile cells along with them and allows migrating cells to influence each other to ensure appropriate cell distribution and shaping of a tissue [29]. Neuroblasts from the SVZ and newly divided neurons are recruited to the striatum following a stroke [30] and neurogenesis and neuronal stem cell migrations occur in the cortex and the hippocampus after ischaemic insults [31] which cause local EFs. Here, we found that the extracellular EF contributes to chain migration of neuroblasts. This new perspective indicates that the development of new therapeutic strategies involving electrical guidance may be useful to treat brain injury and disease.

## Methods and Materials

### Cell Culture

Adult mice (C57BL/6j, 6–8 weeks, male or female), purchased from Charles River Laboratories, were euthanized in a CO_2_ chamber. The method for culturing neurospheres has been described previously [32, 33]. In brief, blocks of SVZ tissue were dissected from mouse brain and digested to release cells. Then a density gradient (OptiPrep density 1.32, Sigma) was used to purify the cells and cultures were suspended in Neurobasal medium (Life technology), supplemented with 2 mM l-glutamine, 2 % B-27 (without Retinyl acetate, Life technology), 20 ng/ml EGF (Life technology), 20 ng/ml FGF-2 (Life technology) and penicillin-streptomycin mixture (Life technologies) at 37 °C in humidified air containing 5 % CO_2._

### Electrotaxis Detection With/Without Extracellular ATP Treatment

Methods of exposing cells to an applied electric field in an electrotaxis detection chamber have been described [34]. The SVZ neuroblasts were seeded in an electrotaxis chamber created in Falcon tissue culture dishes (BD Biosciences) which had been coated with 2 % Matrigel (BD Biosciences) [35, 36]. The cells were allowed to settle and adhere on the base of the chamber for 1 h. A roof of coverglass was applied and sealed with high vacuum silicone grease (Dow Corning Corporation) to the side walls of the chamber [37]. The final dimensions of the shallow chamber, through which the electric current was passed, were 20 mm × 10 mm × 0.3 mm. We applied EFs 10 mV/mm to neurospheres and 5–100 mV/mm to SH-SY5Y cells with/without 100 μM ATP (Sigma) treatment through agar-salt bridges that connected silver/silver chloride electrodes in beakers containing Steinberg’s solution to pools of culture medium at either side of the chamber. The dish was placed on a Zeiss Axiovert 100 microscope with a stage incubator controlling temperature at 37 °C. Images of cells were recorded every 10 min and analyzed with Digital Pixel and MetaMorph imaging systems (Zeiss Axiovert 100 microscope) [18, 37]. Assessment of migration directedness (cosine θ) was used to quantify how directionally a cell migrated in the field, where θ is the angle between the EF vector and a straight line connecting the start and end positions of a cell [23]. Migration rate was analyzed using the following two parameters. Trajectory rate (Tt/T) is the total length of the migration trajectory of a cell (Tt) divided by the given period of time (T). Displacement rate (Td/T) is the straight-line distance between the start and end positions of a cell (Td) divided by the time (T).

### Western Blotting

Cell lysates were collected for Western blot experiments. Western blotting was performed as described previously [38]. Primary antibodies used included anti-β-Catenin (Cell Signalling), anti-N-cadherin (Abcam), anti-P2Y1 (Cell Signalling), anti-p-PKC (Cell Signalling) and anti-GAPDH (Santa Cruz). Immunoblots were detected by Luminata Forte Western HRP substrate (Millipore).

### RNA-Interference

To knock-down P2Y1 in mouse derived neurospheres and SH-SY5Y cells, we used 100 nM of siRNA SMARTpools (a mixture of four siRNA duplexes each) using Dharmafect 1 (Thermo Scientific) according to the manufacturer’s specifications. siRNA^p2y1^ was comprised of: 5′-CUAUUGGUUUUAAUCUGUU-3′, 5′-GUUGAAACUUGUAAAUCUC-3′, 5′-GUAUUUAUUGAAGAGGUUU-3′, 5′- GUACUAGUGUAAAUUCUAU-3′. Under these conditions, 70–90 % of transfection efficiency was achieved, as judged by siGloGreen control (Thermo Scientific). After 72 h transfection, cells were exposed to the EF (no EF application as a control) and cell lysates were collected to confirm the final knock-down effect by Western blotting.

### Immunofluorescent Staining

Cultured neurospheres were fixed with 4 % paraformaldehyde in PBS for 20 min. After incubation with blocking solution for 90 min at 37 °C, cells or tissue sections were incubated overnight with primary antibodies at 4 °C. Primary antibody dilutions used were as follows: Anti-N-Cadherin, 1:200 (Millipore), anti-β-Catenin 1:200 (BD Biosciences) and anti-P2Y1 1:200 (cell signalling). Samples then were incubated for 2 h with a secondary antibody. After washing, counterstaining with DAPI and mounting in anti-fading medium, they were visualized using a Zeiss LSM 510 Meta confocal microscope (Zeiss).

### Brain Slice Culture

SVZ explants were prepared from P7 neonatal mice as described previously [39]. Briefly, brains taken from mice were sliced using a tissue chopper (Mickle Laboratory Engineering)or with a blade under dissecting microscope at 300-500 μm thickness. Then, SVZ tissue was dissected and cut into small pieces under the stereomicroscope to be collected in cold Hanks’ balanced salt solution (Life technology). These pieces were embedded on glass-bottom dishes (Matsunami Glass) in a 3:1 mixture of Matrigel (BD Bioscience) and Neurobasal medium (Life technology) supplemented with 2 mM l-glutamine, 2 % B-27 (Life technology), and penicillin-streptomycin mixture (Life technology) at 37 °C in humidified air containing 5 % CO_2_ [39]. For inhibition experiments, drugs were added to the medium at the beginning of culture and used at the following concentrations: 10 μM digoxin (Calbiochem) and 10 μM ouabain (Calbiochem).

### Microelectrode Measurement

Electrophysiological experiments to measure the electrical field in brain explants were conducted using sharp borosilicate glass microelectrodes. Electrodes were filled with artificial cerebrospinal fluid (ACSF) and had resistances of 2–20 MΩ. All recordings were made at room temperature (20–25 ºC) with an Axoclamp 2A switching amplifier. After balancing the amplifier with respect to ground (0 mV), a pipette was inserted into a 2 × 1 × 0.4 mm brain slice using a Narishige 3D micro-manipulator (Fig. 4b). The brain explants were bathed in ACSF at pH 7.4. The potential difference distribution in the brain slice was determined consistently at 15 s after the electrode was positioned in the tissue and recorded continually for at least 2 min by Scope v3.6-10. At least 4 slices in control and in each treatment group were measured and all experiments were repeated four times. For inhibitor experiments, cells were pre-incubated with 10 μM ouabain or 10 μM digoxin (both are Na^+^/K^+^-ATPase inhibitors, Sigma), for the time indicated and then exposed to the EF.

### Statistical Analysis

A minimum of three replicates was analysed for each experiment presented. Data are presented as the mean ± s.e.m. Student’s *t* test was used to assess the significant difference. Differences were considered as statistically significant with a *p* value <0.05.

## Results

### Extracellular EFs regulate chain migration in explants from the SVZ of mice

**Fig. 1:**
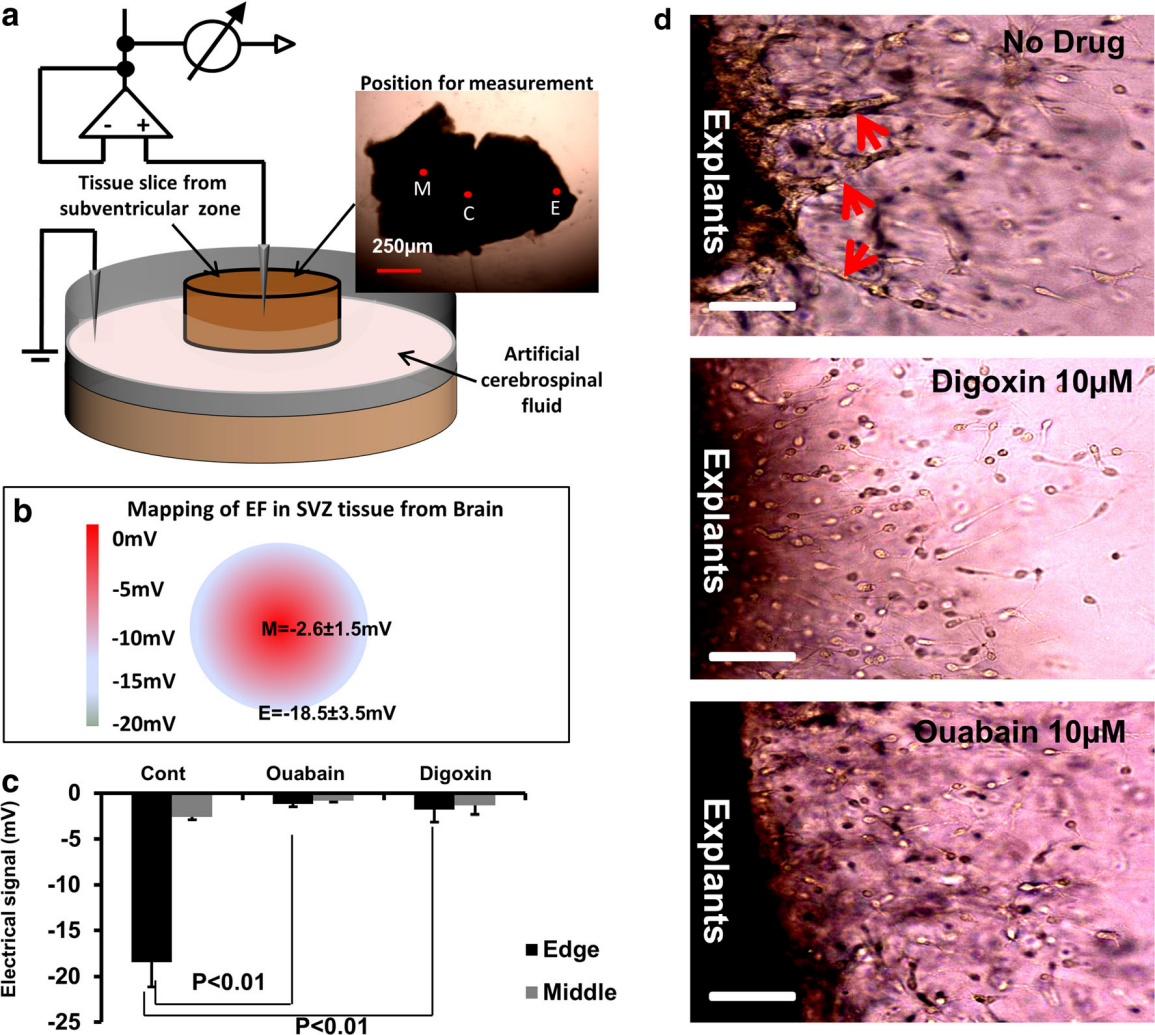
Inhibition of Na^+^/K^+^-ATPase effectively reduced both the endogenous voltage gradient and chain migration of neuroblast in SVZ explants culture. **a** Schematic diagram of the method used to measure the EF in brain slices. **c** (*centre*), M (*middle*) and E (*edge*) represent the points at which the detecting microelectrode measured the extracellular voltage. Reference electrode was located in the medium and connected to earth. **b** Results showed that a voltage drop of −2.6 ± 0.8 and −18.5 ± 3.5 mV were measured at middle (M) and edge (E) of the SVZ slice respectively. The distance between middle point and edge point is 0.4 mm. The voltage gradient between middle and edge is therefore 31.8 ± 4.5 mV/mm. *N* = 5 for each experiment. Triplicate was performed. **c** In explant slices treated with Ouabain or Digoxin, the voltage drops between middle and edge of brain slices were inhibited significantly. The average voltage drop in the middle was reduced from −18.5 mV to −1.2 mV and −1.8 mV by Ouabain and Digoxin respectively. *p* < 0.01. *N* = 5 in one experiment and triplicate was performed. **d** SVZ explants were prepared from p7 mice and cultured for 3 days. In no treatment control (*upper row*), chain migrations formed close to the edge of cultured explant of SVZ as indicated by the red arrows. When the endogenous extracellular EF was inhibited using Digoxin and Ouabain (*middle and lower panel*), cells migrating out from explants did so individually, rather than migrating by forming cellular chains, as was seen in untreated controls (*top panel*). Bar is 100 μm

Neuroblasts migrate as chains in SVZ explants cultured in 3D Matrigel [40]. In addition, endogenous voltage gradients were measured in cultured rat hippocampal slices, with extracellular EFs ranging from 6 to 31 mV/mm, (mean = 17 ± 2.9 mV/mm) [41]. Here we used the glass microelectrode method and measured directly an electrical gradient (EF) of −31.8 ± 4.5 mV/mm in cultured SVZ slices in Matrigel (Fig. 1a and b). The concentration of ions in the extracellular space, for example K^+^, Na^+^, Cl^−^ and Ca^2+^, determine the strength of the EF [11]. When the SVZ slices were cultured in low sodium ACSF (data not shown), or exposed to either 10 μM ouabain or 10 μM dioxin (both inhibitors of Na^+^/K^−^-ATPase), the endogenous EF was reduced significantly, by around 50 % (Fig. 1c). This demonstrates that the formation of the EF in brain tissue explants is dependent at least in part on the function of the Na^+^/K^+^ ATPase pump which regulates the distribution of sodium and potassium between the intracellular and extracellular spaces. Meanwhile, we found that chain migration in cultured SVZ explants was suppressed when the endogenous EF was inhibited by Na^+^/K^−^-ATPase inhibitors (Fig. 1d). This highlights that the endogenous EF may be one element capable of regulating neuronal migration in chains within SVZ explants.

### Mimicking the endogenous electrical cue induced chain migration in cultured mouse neuroblasts

**Fig. 2:**
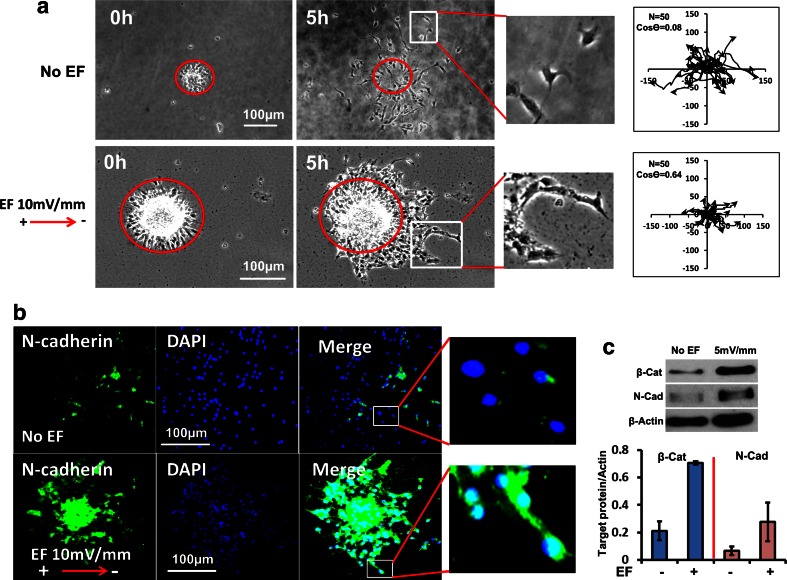
An applied physiological EF induced chain migration in neurospheres. **a** Neurospheres from mouse SVZ were cultured in electrotaxis chambers and an EF of physiological strength (10 mV/mm) was applied for 5 h. In upper row - no EF control, neuroblasts migrated in random directions and did so largely separated and scattered independently from each other (see enlarged image). Lower row - EF applied with cathode to the right. EF-stimulated neurones remained closely adherent to each other and migrated together in chains (see enlarged image at right). Tracks of neuroblast migration are shown in the plots at right. The migration of individual cells is plotted starting at the centre and lines radiate out to show the path and the end point of migration at 5 h. Axes are in μm. N is cell number and Cos θ represents the directedness of cell migration. If Cos θ = 1, it means all cells have migrated directly to the cathode. Control plots (no EF - upper right) show that migration occurs in all directions, randomly. EF-exposed plots (lower right): by contrast migration was directed cathodally (*to the right*). **b** Neurospheres were seeded in electrotaxis chambers with/without an applied EF for 5 h and then the cells were fixed and stained immunofluorescently with an N-cadherin antibody. Cultures exposed to a very low physiological EF of 10 mV/mm (*lower row*) show strikingly enhanced N-cadherin staining compared to no EF controls (*upper row*). **c** A very low physiological EF (5 mV/mm) increased the expression of N-cadherin and β-catenin as determined by Western blotting (3 h exposure to EF). Actin is a loading control. The ratio of protein expression/actin is shown below the corresponding western blots

Chain migration is dynamic; cells within a chain extend and retract filopodia making repeatedly sustained, broken and re-established cell contacts over short time frames [42, 43]. When neurospheres were cultured in a dish, migration of neuroblasts was evident. Without growth factors and with Matrigel coating, neurones migrated away from the spheres randomly and independently with multi-polar morphologies over 5 h (Fig. 2a upper panel, Movie S1). By contrast, when exposed to an applied EF of physiological strength, migrating cells had a simple bipolar morphology and formed chains of cells extending from the spheres (10 mV/mm, Fig. 2a lower panel, Movie S2). The directedness of cell migration (Cos θ) increased significantly from 0.08 ± 0.1 to 0.64 ± 0.03 (*N* = 50; *p* < 0.01) in an applied EF of only 10 mV/mm, indicating strong cathodally-directed migration. In addition, the migration speed at 10 mV/mm showed no significant difference between EF and no EF control (Fig S1A). In short, the applied physiological EF clearly induced chains of cells to form and directed migration to the cathodal side in mouse neurospheres.

Next, we detected two molecules known as markers of typical chain migration in neuroblasts, N-cadherin [5] and β-catenin. Up-regulation of N-cadherin and β-catenin was seen only within the migrating chains exposed to an EF (Fig. 2b). In contrast, there was significantly lower expression of N-cadherin and β-catenin in no EF treated control cells (Fig. 2b). Protein analysis with Western blotting confirmed this (Fig. 2c), indicating that an applied physiological EF induced the formation of chain migration in cultured mouse neuroblasts by increasing cell-cell interaction. This was achieved by an EF-induced increased expression of N-cadherin and β-catenin which promoted and maintained cell-cell contacts and the extension of processes over the other cells in the migrating chains.

### A physiological EF increased cell-cell contacts and directed migration in SH-SY5Y cells

**Fig. 3:**
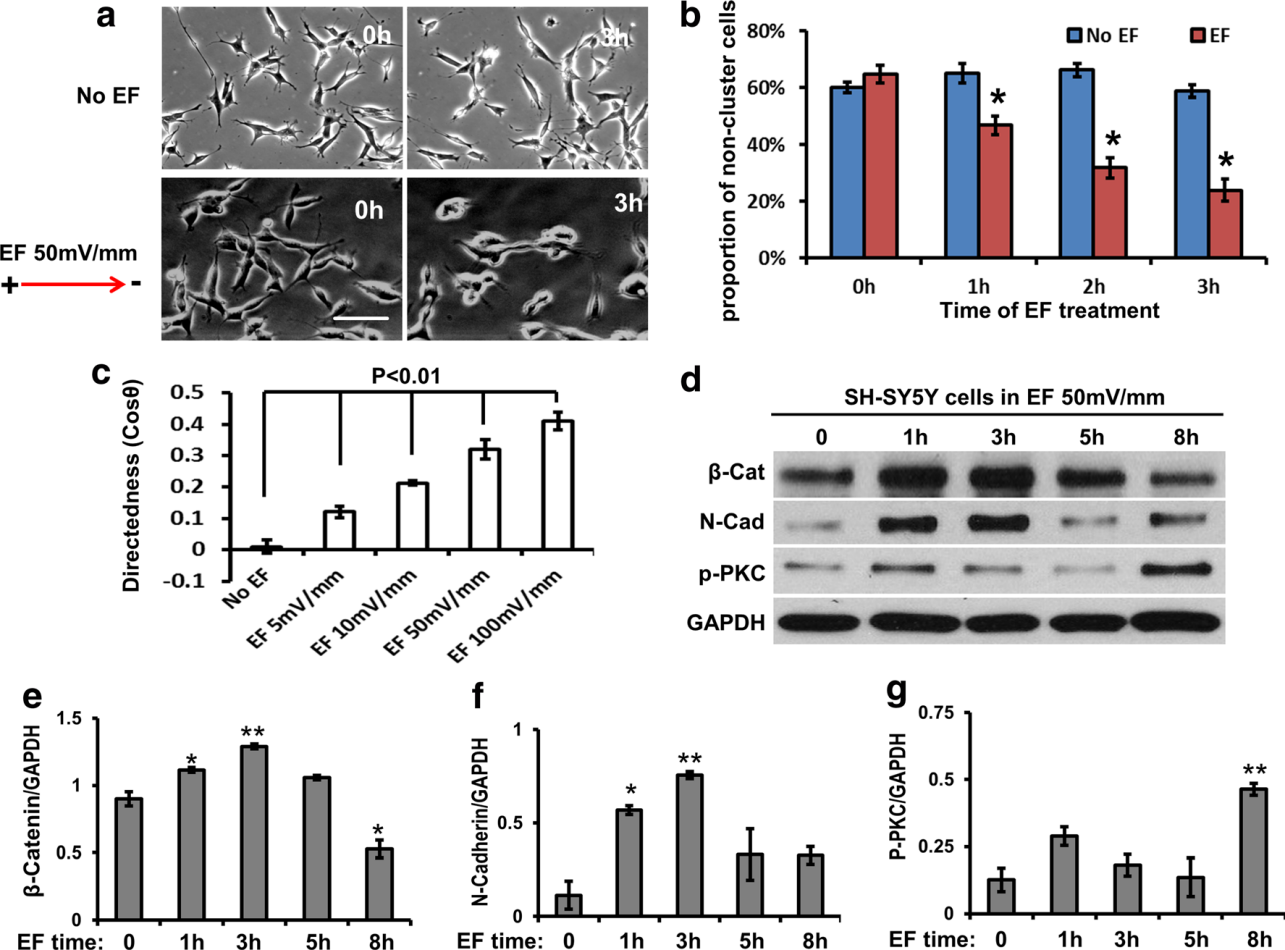
An applied physiological EF induced cell clustering and chain migration in SH-SY5Y. **a** An applied EF of 50 mV/mm (*lower panels*) significantly induced cells to cluster together and to migrate to the cathode. Upper panel – control (no EF); lower panel EF cathode at right. Bars: 20 μm. **b** Cluster formation of SH-SY5Y cells was quantified by counting the percentage of single cell, or groups with less than 3 cells connected together. The reduction of these groups over 3 h in the EF indicates the continuing onset of cell cluster formation. The EF therefore significantly reduced the proportion of single cells due to more cell clusters having formed (>3 cells connection). **c** The directedness (Cos θ) of SH-SY5Y cell migration was field strength dependent, showing significant increases from 5 mV/mm to 100 mV/mm. **d** Western blotting showed that the applied EF effectively increased the expression of N-cadherin and β-catenin at 1 h of EF exposure. The concentration of p-PKC was increased significantly after 8 h treatment by an applied EF. GAPDH is a loading control. (E, F and G) The ratio of protein expression/GAPDH is shown below the western blots for β-Catenin (E), N-cadherin (F) and p-PKC (G)

SH-SY5Y is a human-derived cell line from neuroblastoma and resembles immature sympathetic neuroblasts in culture [44]. Here, we used this cell line to confirm and augment the data from mouse neurospheres. Firstly, we checked cell migration and found that an EF of 50 mV/mm induced significant cell cluster formation and directed migration to the cathode in SH-SY5Y cells (Fig. 3a to c, Movie S3 and S4). We further analysed quantitatively cell cluster formation in SH-SY5Y cells induced by a physiological EF by counting the proportion of single cells or of cell groups with less than 3 cells connected in a real-time recording stack. A reduction of single cells or groups with less than 3 cells connected would demonstrate an increase in cell-cell contacts and collective migration (chain migration). The results showed that an EF of 50 mV/mm significantly reduced single cell numbers and encouraged cell clustering and that these effects increased with longer exposure to the EF (Fig. 3b). In addition, the directedness (Cos θ) of collective cell migration was increased significantly and was dependent on both EF strength (from 5 to 100 mV/mm) and the duration of EF treatment (Fig. 3c). θ is the angle between a line connecting the beginning and end points of cell migration and the X axis. The average directedness of random migration would be 0. The average directedness value would tend to 1 in fully directed migration [25]. Furthermore, our data showed that an applied EF significantly promoted the expression of β-catenin and N-cadherin within one hour and lasting for up to 5 h in SH-SY5Y cells (Fig. 3c, e and f). Since we had shown that the cell-cell adhesion molecules β-catenin and N-cadherin are up-regulated by an applied EF in cultured mouse neurospheres (Fig. 2c), these results are consistent. In this experiment, the applied EF effectively increased cell-cell connections and the cathode directedness of SH-SY5Y cell migration at a physiological EF strength. This indicates that an EF induced chain migration in SH-SY5Y cells. Interestingly, at 8 h in an applied EF the expression of β-catenin and N-cadherin reduced significantly (Fig. 3d, e and f). This indicates that the EF-induced increase in the expression of β-catenin and N-cadherin is a short time function which enhances the maintenance of chain migration in vitro. For long term directed migration in EF (>5 h), other mechanisms, e.g. activation of PKC (Fig. 3d and g), may play a functional role.

### P2Y1 Mediated the Chain Migration Induced by a Physiological EF

**Fig. 4:**
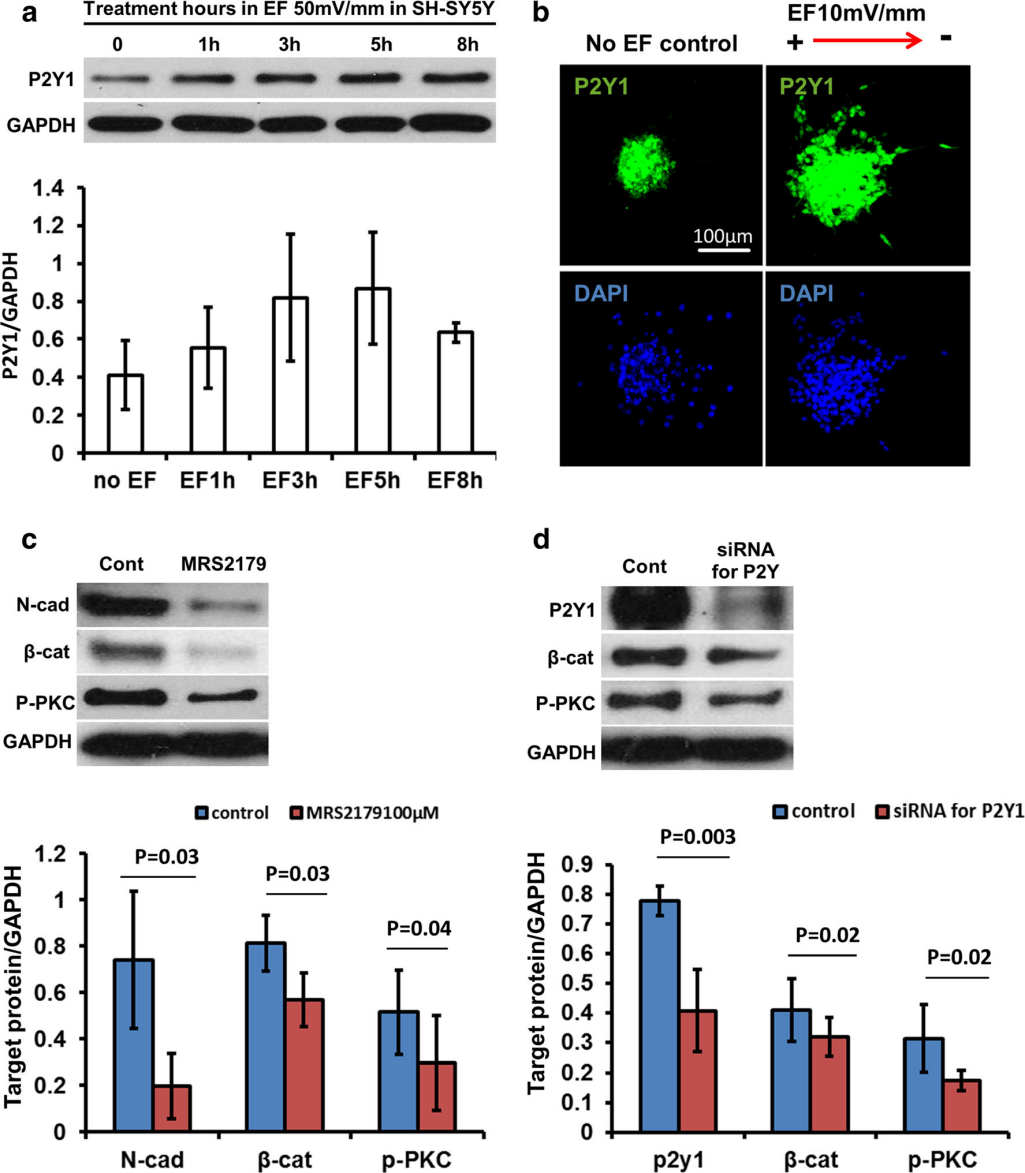
P2Y1 mediated neuronal chain migration induced by a physiological EF. **a** Western blotting showed the P2Y1 band at 45KD to be up-regulated in a time dependent manner by a physiological EF, with an increase evident within 1 h. **b** Neurospheres from mouse SVZ were seeded in electrotaxis chambers with/without an applied EF for 5 h and then cells were fixed and stained immunofluorescently with P2Y1 receptor antibody. The applied EF significantly increased the expression of P2Y1 in the cells which migrated out from the cultured mouse neurosphere. **c** Inhibition of P2Y1 receptors with a specific inhibitor (100 μM MRS2179) significantly reduced the expression of N-cadherin and β-catenin and blocked the activation of PKC using western blotting and mouse neurospheres. **d** Inhibition of P2Y1 receptors with siRNA also effectively inhibited the expression of N-cadherin and activation of PKC. Graphs below C and D demonstrate corresponding quantification. All experiments were triplicate

We have reported that an applied EF increases cathodally directed migration in mouse neuroblasts and that this is mediated by P2Y1 receptors [24]. Atypical protein kinase C (PKC) is required to establish and control cell polarity [45, 46]. In addition, we have reported that PKC played a key role in cell polarity and directed cell migration induced by an applied EF [25, 47]. Inhibition of PKC in RMS neuroblasts disrupts their ability to reorient the centrosome and stabilize processes, and so leads to failure of directed neuronal migration [48]. Here, we found that an applied EF increased the expression of P2Y1 receptors significantly and in a time dependent manner using both western blotting in SH-SY5Y cells and immunofluorescent staining in mouse neuroblasts (Fig. 4a and b). Furthermore, inhibition of P2Y1 receptors with MRS2179 and siRNA had similar effects in inhibiting the expression of N-cadherin, β-catenin and activation of PKC (Fig. 4c and d). This indicates that the chain migration induced by an applied EF in neuroblasts may be mediated by the P2Y1 receptor.

### Extracellular ATP Increased EF-Directed Neuronal Migration

**Fig. 5:**
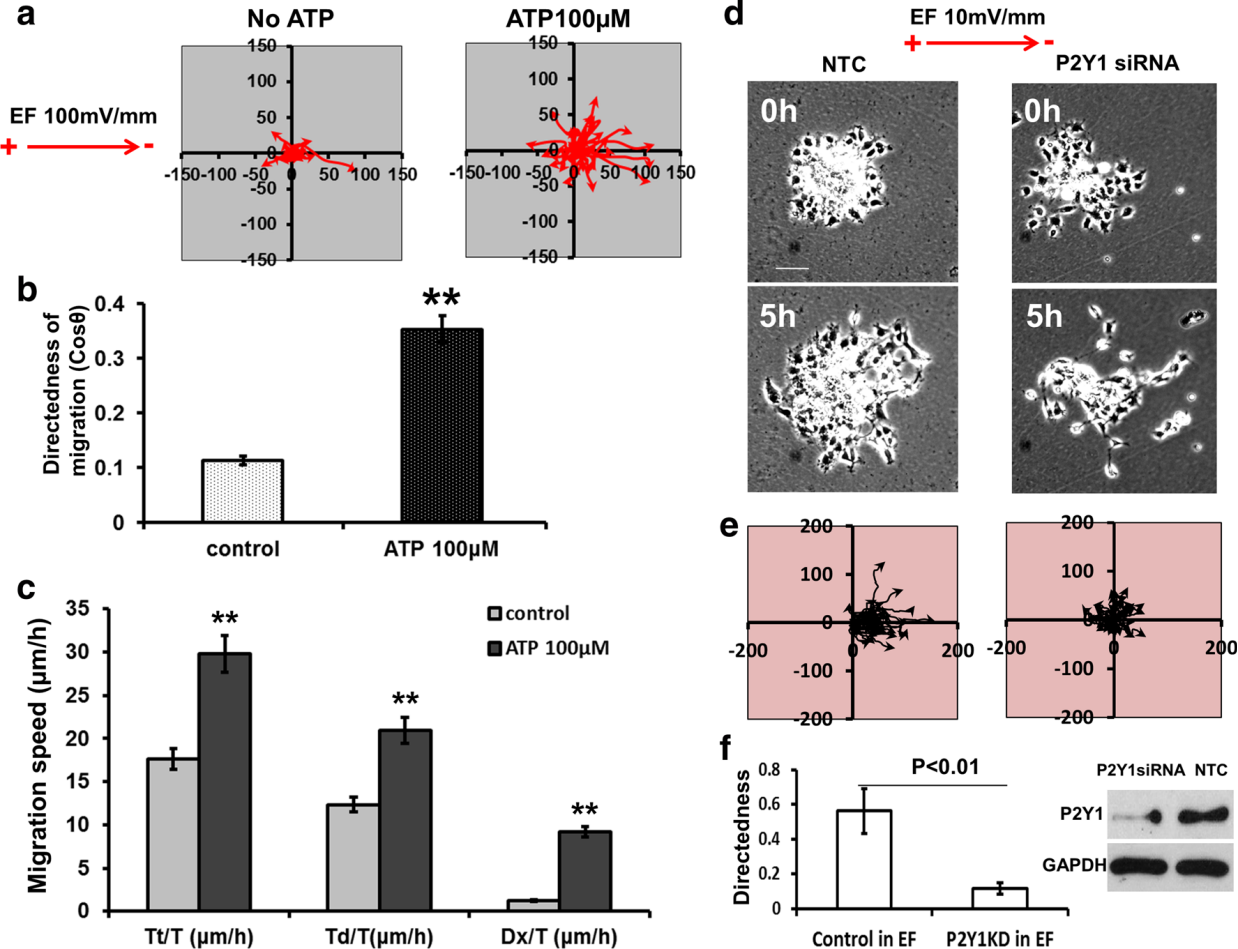
Extracellular ATP increased the directed migration of SH-SY5Y cells and knock down of P2Y1 receptors inhibited directed migration in mouse neurospheres. **a** to **c** In SHSY5Y cells (human), 100 μM ATP significantly increased the directedness (Cos θ) of cells migration induced by an applied EF from 0.11 to 0.35 (EF = 100 mV/mm, *n* = 200). The migration speed including Trajectory rate (Tt/T), Displacement rate (Td/T) and Displacement speed along the x-axis (Dx/T) also increased significantly: Tt/T from 17.6 ± 1.2 to 29.7 ± 2.1 μm/h, Td/T from 12.3 ± 0.9 to 20.9 ± 1.5 μm/h, Dx/T from 1.2 ± 0.09 to 9.2 ± 0.6 μm/h. If cells migrated preferentially towards the right, an average directedness would be larger than 0 and approaching 1. Trajectory rate (Tt/T) is the total length of the migration trajectory of a cell (Tt) divided by the given period of time (T). Displacement rate (Td/T) is the straight-line distance between the start and end positions of a cell (Td) divided by the time (T). Displacement speed along the x-axis (Dx/T) is a cell’s displacement distance along the x-axis (Dx) divided by the time (T). **p < 0.01. **d** to **f** In cultured mouse neurospheres, knock down of P2Y1 receptor with siRNA (right column in D) significantly inhibited the directedness of cell migration compared with no siRNA control (left column in D). In E, the diagram of line plots showed cathodally directed cell migration to be reduced by inhibition of P2Y1 receptors with siRNA. Directedness (Cos θ) dropped from 0.56 to 0.12 (*n* = 30 ~ 35, *p* < 0.01). n is cell number. No EF control is shown in Fig. 2a. The western blot (right in F) shows down-regulation of P2Y1 receptor expression by siRNAP2Y1. NTC is no transfection control. GAPDH is loading control. All experiments were triplicate

P2Y1 is a receptor for extracellular ATP which acts as a neurotransmitter and neuromodulator in the CNS. For example, ATP induces increases in calcium and neuronal excitation in various brain regions [49–52]. To confirm a functional role of the P2Y1 receptor in EF-induced chain migration of neuroblasts, we next observed the migration of mouse neurospheres when P2Y1 was either activated or inhibited using extracellular ATP, or the specific inhibitor MRS2179 in SHSY5Y cells. We found that extracellular ATP (100 μM) markedly increased both the directedness (from 0.11 ± 0.01 to 0.35 ± 0.03; *p* < 0.01) and the migration speed (Tt/T) of SHSY5Y cells (from 17.6 ± 1.2 μm/h to 29.7 ± 2.1 μm/h; *p* < 0.01) in a physiological EF of 100 mV/mm (Fig. 5a to c). Furthermore, when P2Y1 receptors were inhibited with siRNA in mouse neuroblasts (neurospheres), the directed migration induced by an EF was inhibited profoundly (directedness from 0.56 to 0.12; Fig. 5d – f). This suggests that EF regulated chain migration of neuroblasts may be mediated by the P2Y1 extracellular ATP receptor.

## Discussion

In adult brain, neuroblasts are generated and complete their initial differentiation in the SVZ. They then migrate directionally as neuronal chains, sliding along each other in the RMS from SVZ to OB [53–55]. Upon arriving at the OB, the new neurons differentiate into olfactory interneurons, and integrate into the olfactory processing system. Chain migration is regulated by multiple cellular and molecular cues, the coordination of which is still unclear [4, 10, 56]. We report a novel mechanism in which extracellular electrical signals contribute to directed chain migration of neuroblasts through P2Y1 receptor signalling.

Early recordings showed that the cortical surface was 0.5 to 5.5 mV positive to the ventricle [57]. In addition, synchronous neuronal discharges within highly laminar structures such as the hippocampus generate substantial extracellular field potentials [41]. Turner et al. found extracellular EFs ranging from 6 to 31 mV/mm in adult rat hippocampus, whilst EFs ranged from 13 to 43 mV/mm in adult turtle cerebellum [58]. In the developing embryonic neural tube also, extracellular EFs of 10–100 mV mm^−1^ have been recorded [13, 15, 16, 59]. Here, we detected an extracellular EF of 31.8 ± 4.5 mV/mm in neonatal mouse SVZ slices (Fig. 1). Previously, we have measured directly an electric gradient of 5.7 ± 1.2 mV/mm in the extracellular spaces along the rostral migratory stream [24]. Clearly extracellular electrical gradients are widespread in different developing, neonatal and adult brain locations although their physiological roles remain little recognised and poorly understood.

EFs are widespread also in other developing and regenerating tissues where they regulate cell division and directed cell migration [11, 12, 25, 38]. Intriguingly, most cell migrations in developing and damaged brain occur through tissues in which steady electrical signals exist [11]. For example, epileptic seizure, stroke, ischaemia, migraine and acute damage to the hippocampus all induce extracellular electrical signals in brain that persist for hours [19–23]. Thus, the endogenous EFs represent a novel and powerful signalling mechanism with the potential to guide cell migration. In addition, EF-induced directional re-orientation of the leading edge cells of large epithelial sheets is E-cadherin dependent [60]. To migrate at high speed over long distance within neuronal chains, the collective of neuroblasts needs to slide along each other efficiently [61] and for this, a consistent direction and maintained cell-cell contacts are essential. Here, we found that a physiological EF induced cathodal-directed migration and increased the expression of N-cadherin and β-catenin to promote and maintain the cell-cell connections between neuroblasts. Consistent with this, inhibition of the extracellular electrical signal in SVZ slices effectively disrupted the formation and directed migration of neuroblast chains. This is the first evidence that the natural extracellular electrical gradients of the brain contribute to the formation, maintenance and directed migration of chains of neuroblasts.

Several proteins that regulate chain formation in RMS have been identified. For example, mice lacking the cell-surface receptors ErbB4 and ApoER2 lost chain migration in the RMS and new neurons arriving in the OB were reduced [62, 63]. Extracellular matrix-related molecules such as α6β1-integrin and ADAM2 protease also are involved [64–66], indicating that both cellular receptor and extracellular matrix elements regulate neuroblast chain formation in brain. Additionally, extracellular signals such as ATP dynamically reorganize the cytoskeleton of each migrating neuroblast and regulate chemotaxis of microglia via Gi/o-coupled P2Y receptors [67, 68]. Early studies showed that electrical stimulation of axons liberated ATP [69, 70] and that extracellular ATP induces excitation and increases in calcium in neurons [49, 50, 71, 72], Erk activation [73], and calcium wave propagation [74]. This calcium signalling is essential for directed cell migration induced by an applied EF [75]. Furthermore, an applied EF transiently elevated extracellular ATP and caused Akt phosphorylation that was additive to insulin and inhibited by suramin (inhibitor of P2Y receptors) [76]. Tran et al. demonstrated a role for extracellular ATP, purinergic receptors and protein kinase signalling in enhancing N-cadherin expression indicating a role in cell-cell interactions [77]. We found that an applied electrical signal which mimicked that found in brain up-regulated the expression of P2Y1 receptors to increase expression of N-cadherin and β-catenin. Therefore, the mechanism of EF-induced chain migration of neuroblasts most likely involves activation of the ATP/P2Y1 signalling pathway through up-regulation of P2Y1 expression and increased release of ATP.

Increased neurogenesis and migration of progenitor cells have been observed in animal models of epilepsy, stroke, trauma, Alzheimer’s disease, Parkinson’s disease and Huntington’s disease [78]. In addition, high expression of P2Y1 is required for neuroblast migration in brain [68]. We found that the exogenous EF promoted chain migration of neuroblasts through increasing the expression of P2Y1. This indicates that it may be possible to deliver neuroblasts to sites of brain injury and disease by directing their migration using an applied DC electric field. EFs have been used clinically in the treatment of human spinal cord lesions [79] and extending their use to brain lesions therefore is feasible.

## Conclusions

We showed previously that there is an extracellular EF between SVZ and the OB which contributes to guidance of neuroblast migration along the RMS [24]. Here, we show further that this endogenous EF regulates chain migration of neuroblasts by promoting and maintaining cell-cell connections by up-regulating the expression of N-cadherin and β-catenin. In addition, we found that up-regulation of the P2Y1 receptor contributes to EF-induced chain migration. Our data indicate that this naturally occurring electrical gradient within the extracellular spaces of the RMS acts as a guidance cue directing chain migration of neuroblasts between the SVZ and the OB. These findings present a highly novel perspective of one of the mechanisms controlling and guiding neuroblast migration in a specific part of the mammalian brain.

## Electronic supplementary material

Below is the link to the electronic supplementary material.

ESM 1 (DOC 214 kb)

Movie S1 (AVI 1615 kb)

Movie S2 (AVI 1299 kb)

Movie S3 (AVI 517 kb)

Movie S4 (AVI 389 kb)

Movie S5 (AVI 2074 kb)

Movie S6 (AVI 1612 kb)

## Abbreviations

EF: Electrical field
SVZ: Subventricular zone
P2Y1: P2Y purinoceptor 1
RMS: Rostral migratory stream
OB: Olfactory bulb
CNS: Central nervous system
CSF: Cerebro-spinal fluid
miR-9: Brain-specific microRNA-9
PKC: Protein kinase C
