# Supplementary material for: Physiological Electrical Signals Promote Chain Migration of Neuroblasts by Up-Regulating P2Y1 Purinergic Receptors and Enhancing Cell Adhesion
Source: Stem Cell Rev. 2014 Aug 7;11(1):75–86. doi: 10.1007/s12015-014-9524-1 (PMC4333314; doi:10.1007/s12015-014-9524-1)
Supplement: Supplementary file 1 — (DOC 214 kb) [file 12015_2014_9524_MOESM1_ESM.doc]

**Supplemental Information**

**Electrical signals in brain promote chain migration**

**of neuroblasts by up-regulating P2Y1 purinergic receptors**

Lin Cao^1^, *, **^#^,** Jin Pu^1^, **^#^,** Roderick H Scott^1^, Jared Ching^2^, Colin D McCaig^1^*

^1^ School of Medical Sciences, Institute of Medical Sciences, University of Aberdeen, Aberdeen, UK

^2^  Department of Neurosurgery, Aberdeen Royal Infirmary, Aberdeen AB25 2ZD

* **Corresponding author:** Prof. Colin D McCaig, School of Medical Sciences, University of Aberdeen, Aberdeen AB25 2ZD, UK. Tel: 44 (0)1224 437394, Fax: 44(0)1224437465, E-mail: [c.mccaig@abdn.ac.uk](mailto:c.mccaig@abdn.ac.uk)

Dr. Lin Cao, School of Medical Sciences, University of Aberdeen, Aberdeen AB25 2ZD, UK. [Tel: 44 (0)1224 437532](mailto:Tel: 44 (0)1224 437532), Fax: 44(0)1224 437465, E-mail: [L.cao@abdn.ac.uk](mailto:L.cao@abdn.ac.uk)

**# Contributed equally**

**Supplemental Figure and legend**


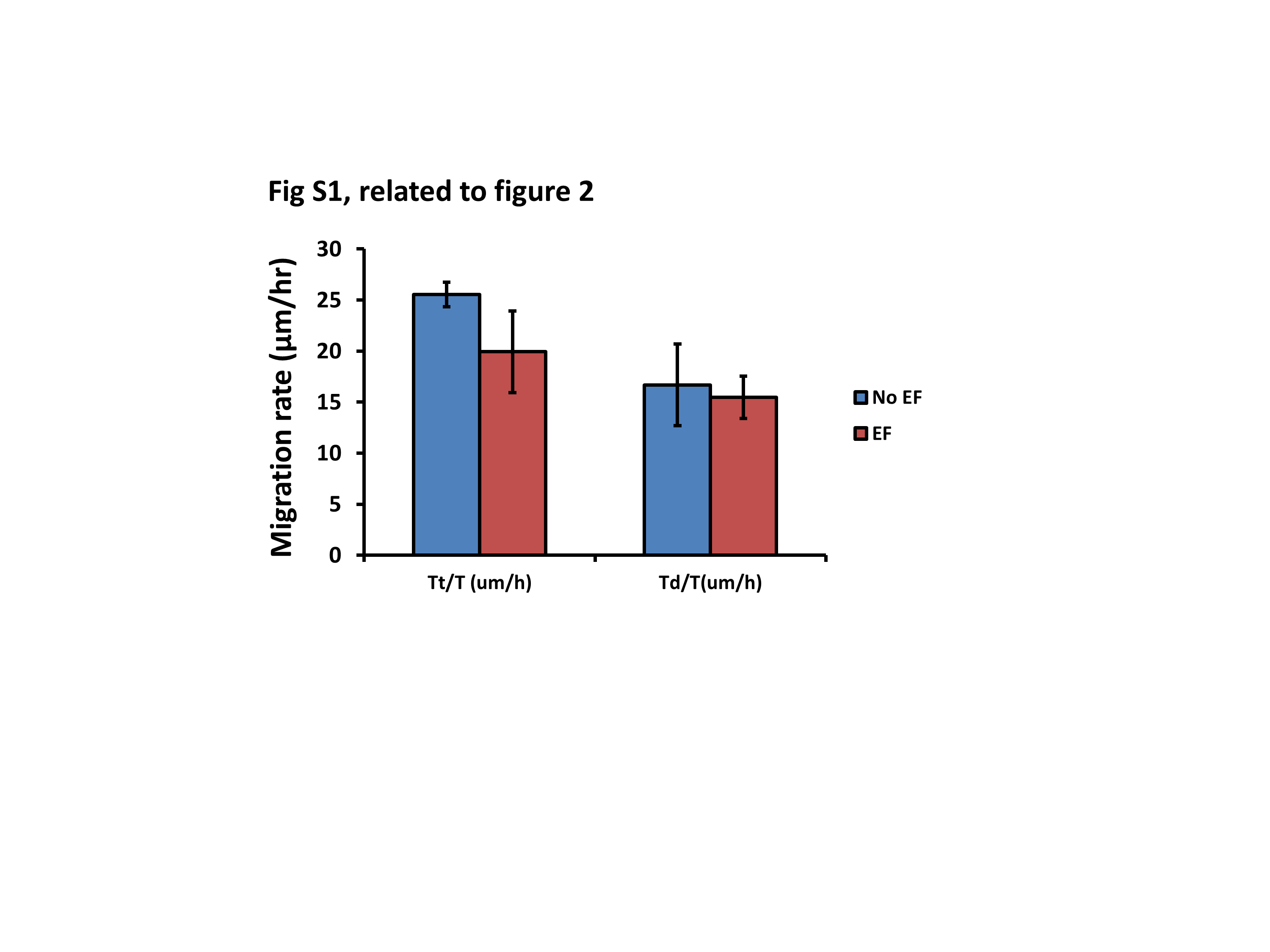


**Figure S1. Migration rate of neuroblasts from mouse neurosphere with/without electric fields.** Related to figure 2. Trajectories of cell migration show the directional migration of SVZ neuroblasts towards the cathode (the right) for a period of 5 hours. An electric field which is as small as 10mV/mm did not enhance the migration rate. The data were grouped from 84-96 cells from three or more independent experiments. The data are shown as mean ± S.E.M.

**Supplemental Movies legends**

**Movie S1. Neuroblasts from mouse cultured neurosphere migrated in random direction and did not show the chain formation without an applied EF.** Related to figure 2. Cell migration was recorded with 10-min interval for 5 hours.

**Movie S2. Neuroblasts from mouse cultured neurosphere showed a directed migration to cathode and the chain formation in an applied EF.** Related to figure 2. Cell migration was recorded with 10-min interval for 5 hours. A 10mV/mm EF was applied.

**Movie S3. SH-SY5Y cells migrated in random direction without an applied EF.** Related to figure 3. Cell migration was recorded with 15-min interval for 5 hours.

**Movie S4. SH-SY5Y cells migrated to cathode and tend to form clusters in an applied EF.** Related to figure 3. Cell migration was recorded with 15-min interval for 3 hours. A 50mV/mm EF was applied.

**Movie S5. Inhibition of P2Y1 with siRNA in Neuroblasts from mouse cultured neurosphere to show a random migration without an applied EF.** Related to figure 5. Cell migration was recorded with 10-min interval for 5 hours.

**Movie S6. Inhibition of P2Y1 with siRNA in Neuroblasts from mouse cultured neurosphere to show a reduced directed migration and chain formation in an applied EF.** Related to figure 5. Cell migration was recorded with 10-min interval for 5 hours. A 10mV/mm EF was applied.
